# Radical prostatectomies in Austria, 1997–2004

**DOI:** 10.1186/1756-0500-1-48

**Published:** 2008-07-21

**Authors:** Gerald Haidinger, Stephan Madersbacher, Georg Schatzl, Christian Vutuc

**Affiliations:** 1 Department of Epidemiology, Centre of Public Health, Medical University of Vienna, Borschkegasse 8a, 1090 Vienna, Austria; 2 Department of Urology and Andrology and Ludwig Boltzmann Institute for Urological Oncology, Donauspital – SMZO, Langobardenstrasse 122, 1220 Vienna, Austria; 3 University Clinic for Urology, Medical University of Vienna, Währinger Gürtel 18-20, 1090 Vienna, Austria

## Abstract

**Background:**

The introduction of PSA testing in Austria led to a steep increase of the incidence of prostate cancer. We want to present the course of the number of newly diagnosed cases of prostate cancer in Austria since 1997, and set these numbers in relation to the total of radical prostatectomies (with resection of lymph nodes) in the same time period. All numbers were retrieved from health statistics of Statistics Austria. The report period of cancer cases and of RPE comprises the years 1997–2004. All calculations were performed for totals as well as for 5-year age groups (40–89 years of age).

**Findings:**

The number of prostate cancer cases rose from 1997 to 2004 by 35%, while the number of RPE rose by 94% in the same time period. The proportion of RPE in relation to new cases rose from 41% in 1997 to 59% in 2004.

**Conclusion:**

A slight decrease of prostate cancer mortality can already be observed in Austria, but the question of over-treatment still awaits analysis.

## Findings

Opportunistic Prostate-Specific-Antigen testing (PSA) of healthy men started at the beginning of the 1990s and led to a steep increase of prostate cancer incidence [1-3]. In Austria data on surgical treatment of prostate cancer are available since 1997. The purpose of this paper is to present the correlation between the number of newly detected cases and the number of radical prostatectomies with resection of lymph nodes (RPE) by 5 year age group.

Data on the number of newly detected cases and number of prostatectomies where obtained from Statistics Austria. The data on radical prostatectomies by age group are available since 1997 only. The data set covers the age group 40 to 89 years comprising a population of 1.738,655 men in the year 2000. Since cases are anonymized it is indeterminable whether newly detected cases are operated in the year of diagnosis or in the following year, if ever.

Cases treated by perineal cryosurgery were excluded, due to the small total number (0 to 2 cases per year).

The number of newly detected cases and of RPE (1997 through 2004) as well as the calculated percentage per year by 5 year age group are given in table 1. The absolute number of prostate cancer cases rose from 3999 in 1997 to 5416 in 2004 (+35.4%, all age groups). The absolute number of RPE rose from 1648 in 1997 to 3200 in 2004 (+94.2%, all age groups). The percentage of RPE in relation to the number of newly detected cases rises from 41% in 1997 to 59% in 2004 (all age groups). The increase of this percentage is observed in all age groups younger than 70 years but not in older age groups, the linear trend over time is significant for all age groups and for totals, except for age group 40–44 years.

**Table 1:** Number of newly detected cases of prostate cancer in Austria, and number of radical prostatectomies 1997–2004, as well as proportion of RPE/incidence, and P value for linear time trend, totals and 5-year age groups of men aged 40–89.

Age	1997			1998			1999			2000			2001			2002			2003			2004			
	Nndc	RPE		Nndc	RPE		Nndc	RPE		Nndc	RPE		Nndc	RPE		Nndc	RPE		Nndc	RPE		Nndc	RPE		

	n	n	%	n	n	%	n	n	%	n	n	%	n	n	%	n	n	%	n	n	%	n	n	%	P-value

40–44	3	4	>100	5	4	80	5	4	80	16	14	87	18	15	83	17	18	>100	22	17	77	25	25	100	0.338
45–49	26	21	81	40	37	92	44	35	80	74	71	96	85	81	95	95	87	92	125	123	98	109	118	>100	< 0.001
50–54	127	99	78	149	118	79	166	157	95	199	171	86	256	240	94	266	259	97	267	279	>100	254	258	>100	< 0.001
55–59	371	300	61	440	382	87	496	433	87	522	482	92	541	518	96	559	492	88	597	552	92	585	612	>100	< 0.001
60–64	490	369	75	528	359	68	589	454	77	784	688	88	907	798	88	995	884	89	1158	1067	92	1105	980	89	< 0.001
65–69	841	515	61	880	568	65	924	560	61	938	666	71	962	685	71	910	651	72	1065	842	79	1011	789	78	< 0.001
70–74	837	299	36	873	288	33	976	288	30	1042	355	34	1035	390	38	998	370	37	1074	500	47	1001	377	38	< 0.001
75–79	554	34	6	655	31	5	737	38	5	698	40	6	674	42	6	644	26	4	732	119	16	718	36	5	< 0.001
80–84	383	4	1	300	2	<1	316	0	0	333	1	<1	328	2	<1	339	0	0	403	38	9	367	2	<1	< 0.001
85–89	367	0	0	348	0	0	340	0	0	317	1	<1	322	1	<1	258	0	0	284	15	5	325	1	<1	< 0.001

Total	3999	1648	41	4218	1826	43	4593	1969	43	4923	2489	51	5128	2774	54	5081	2791	55	5727	3167	55	5416	3200	59	< 0.001

Nndc ... Number of newly detected cases.

RPE ... Radical Prostatectomy (with resection of lymph nodes).

P-value ... P value for linear time trend.

The increase of the incidence of prostate cancer is well documented for Austria [1,2], however the absolute numbers are for the first time set in relation to the number of RPE nationwide. Within 8 years, the total number of RPE increased by 94.2%, thus nearly doubled, with the increase mainly occurring in the age group 55–69 years. Starting at age 70 years the percentage of RPE remains more or less stable on a substantially lower level.

The number of RPE we used in our calculations represents the official hospital discharge statistics of Austria comprising of the collected data of all hospitals (including private hospitals), but excludes a very small number of self-pay patients. Thus our results negligibly may underestimate the relation of RPE to the number of newly detected cases. A further limitation of our analyses is caused by legal constrains: due to data protection laws we are limited to the use of depersonalized data.

The increase of RPE in Austria can be explained by at least three factors: a) operation technique meliorated significantly, now is a standard procedure, perioperative mortality is low and the risk of postoperative morbidity such as incontinence and erectile dysfunction decreased in Austria [4,5], consequently acceptance of RPE in patients increased. b) By the introduction of PSA testing the target group of prostate cancer screening shifted towards younger age groups, meaning that carcinoma more frequently is sought in younger men [3]. c) Because of the slow progression of most cases, RPE only makes sense when health status permits RPE and patients have a life expectancy of at least 10 years [6].

The high percentage of RPE in men younger than 70 years surely also is influenced by the fact that counselling (towards RPE or radiation therapy) in Austria is performed by urologists. In this context, it is worth mentioning, that the primary intention of this manuscript is to provide the data which can serve as a basis for a constructive discussion about counselling and the provision of the most adequate therapeutic regimes.

The reason for the more or less stable percentage in men aged 70 years or older may lie in the fact that therapy of prostate cancer in these patients more often relies on watchful waiting, active surveillance, external beam radiation, brachytherapy or hormonal treatment. Detailed data for these therapies are not available.

In conclusion, currently 59% of prostate cancer cases detected are treated by RPE, a percentage steadily rising from 1997 on. On one hand, the dramatic increase of RPE already leads to a slight reduction of prostate cancer mortality in Austria [3,7], on the other hand the question of over-testing and over-treatment, and added morbidity remains unanswered and analyses are still pending for Austria.

## Competing interests

The authors declare that they have no competing interests.

## Authors' contributions

GH and CV planned the study and drafted the manuscript. CV obtained the data, GH and CV performed the statistical analysis. SM and GS contributed in preparing the manuscript. All authors read and approved the final manuscript.
